# Mindless Eating Challenge: Retention, Weight Outcomes, and Barriers for Changes in a Public Web-Based Healthy Eating and Weight Loss Program

**DOI:** 10.2196/jmir.2218

**Published:** 2012-12-17

**Authors:** Kirsikka Kaipainen, Collin R Payne, Brian Wansink

**Affiliations:** ^1^VTT Technical Research Centre of FinlandTampereFinland; ^2^Department of MarketingNew Mexico State UniversityLas Cruces, NMUnited States; ^3^Charles H. Dyson School of Applied Economics and ManagementCornell UniversityIthaca, NYUnited States

**Keywords:** Adherence, Barriers, Habits, Internet, Self report, Small changes, Weight loss programs

## Abstract

**Background:**

Most dietary programs fail to produce lasting outcomes because participants soon return to their old habits. Small behavioral and environmental changes based on simple heuristics may have the best chance to lead to sustainable habit changes over time.

**Objective:**

To evaluate participant retention, weight outcomes, and barriers for changes in a publicly available web-based healthy eating and weight loss program.

**Methods:**

The National Mindless Eating Challenge (NMEC) was a publicly available, online healthy eating and weight loss program with ongoing recruitment of participants. This volunteer sample consisted of 2053 participants (mean age 39.8 years, 89% female, 90% white/Caucasian, BMI mean 28.14). Participants completed an initial profiling survey and were assigned three targeted habit change suggestions (tips). After each month, participants were asked to complete a follow-up survey and then receive new suggestions for the subsequent month.

**Results:**

In terms of overall attrition, 75% (1549/2053) of participants who completed the intake survey never returned to follow up. Overall mean weight loss among returning participants was 0.4% of initial weight (*P*=.019). Participants who stayed in the program at least three calendar months and completed at least two follow-up surveys (38%, 189/504) lost on average 1.8 lbs (1.0%) of their initial weight over the course of the program (*P*=.009). Furthermore, participants who reported consistent adherence (25+ days/month) to the suggested changes reported an average monthly weight loss of 2.0 lbs (*P*<.001). Weight loss was less for those who discontinued after 1-2 months or who did not adhere to the suggested changes. Participants who reported having lost weight reported higher monthly adherence to suggestions (mean 14.9 days, SD 7.92) than participants who maintained (mean 12.4 days, SD 7.63) or gained weight (mean 12.0 days, SD 7.50; *F*=14.17, *P*<.001). Common reported barriers for changes included personally unsuitable or inapplicable suggestions, forgetting or being too busy to implement changes, unusual circumstances, and emotional eating.

**Conclusions:**

Because the bulk of the free and commercially available online diet and nutritional tools conduct no evaluation research, it is difficult to determine which aspects of a program are successful and what are reasonable expectations of results. The results of this study suggest that online interventions based on small changes have the potential to gradually lead to clinically significant weight loss, but high attrition from publically available or “free” programs still remains a challenge. Adherence to and effectiveness of small habit changes may be improved through further tailoring to individual circumstances and psychological needs.

## Introduction

Effective healthy eating interventions are needed to reverse the global obesity trend [1]. Most current weight loss programs and diets have failed to produce sustainable changes, partially due to the difficulty of maintaining healthy eating behaviors in an environment that constantly urges people to consume unhealthy food in excess [2]. Furthermore, programs that focus on education about calories and nutritional guidelines may place such high demands on participants’ cognitive abilities that long-term adherence will be difficult [3].

Recent research suggests that small and concrete habit changes that gradually lead towards larger lifestyle changes may be the best way to achieve sustainable results [1]. Habit is starting to be considered as one of the most powerful predictors of eating behavior, and habits are mainly cued by situational factors [4]. Simple heuristics that are applicable in a wide variety of situations can help people to modify their automatic responses to food triggers in their environment to form new healthier habits [5]. In this way, healthful choices become activated by cues in the environment without effortful deliberation, intentions, or willpower [6].

The small-changes approach has been successfully embraced by various individuals and policy makers [1], but the challenge for interventions is to provide easy and effective habit change suggestions for each individual. Tailoring interventions to match individual characteristics and needs can lead to significant improvements in their effectiveness and relevance to recipients [7-9]. Dietary counselors can do tailoring in person-to-person interactions, but the resources for individual counseling are limited. The reach of habit change interventions can be best widened to the general population through partially or wholly automated web-based programs. Web-based weight loss and maintenance programs have demonstrated moderate efficacy in behavioral change [9-11], and randomized controlled trials have shown varying outcomes ranging from no weight loss to an average loss of 16.8 lbs (7.6 kg) [12]. Individualized counseling and feedback appear to improve outcomes [13].

The small-changes approach is still a relatively new concept in web-based intervention programs. To our knowledge, only one online intervention thus far has utilized the approach to support participants in making small sustained changes in dietary or physical activity behaviors [14]. The results of a randomized controlled trial showed that this intervention had positive effects on eating habits and the amount of physical activity, but it was no more effective than generic information [14]. Another online intervention, Daily Challenge, sends participants daily suggestions of small actions to improve well-being [15]. Its impact on well-being has not yet been evaluated.

The aim of this research was to evaluate the retention and weight outcomes of an online, tailored healthy eating and weight loss program, National Mindless Eating Challenge (NMEC), and recognize barriers for small habit changes. The NMEC program provides participants a tailored set of habit change suggestions for each month and offers them a checklist for self-monitoring and accountability [5]. The suggestions are based on findings from laboratory research about eating behavior [16]. Prior pilot trials of the NMEC program indicate that it can result in a slow and steady weight loss through small lifestyle changes that have the potential to become permanent [5].

## Methods

### Intervention

The National Mindless Eating Challenge (NMEC) was a publicly available, Internet-based dietary intervention program designed to aid participants in making small, effective eating-related changes in their daily lives [5]. Multimedia Appendix 1 shows the main page of the program. The program was offered passively from December 2006 until July 2009 as a resource to the public who found the program via search engines or hyperlinks or were directed to the program by a member of the research group as a response to their inquiry for assistance in weight management. The move to a new platform in June 2007 offered a more complete capture of data. This study was conducted with participants who were involved with the program for any period of time between July 2007 and July 2009. Participants who signed up in the freely available program completed an initial survey consisting of self-report measures of demographics, physical characteristics, and psychological characteristics. After completing the survey, they selected their initial eating goals (lose or maintain weight, eat healthier, eat more, or help their family eat better) and subobjectives. They were then randomly assigned three different environmental, behavioral, or cognitive suggestions that were relevant to the eating goal and subobjective they had chosen.

The habit change suggestions were selected from a pool of 232 different research-based suggestions, such as using smaller plates at meals, never eating directly from a package, or drinking water with every meal and snack [16]. The suggestions were phrased in an active form (such as “Put down your utensils between bites”). Some suggestions provided a brief explanation on why the change would work (such as “This will allow you to slow down the pace of your eating”). Additionally, the program contained references to the *Mindless Eating* book [17], which details the underlying research and contains similar suggestions for changing one’s habits and environment.

After receiving the suggestions, participants were asked to estimate their adherence to the changes and how easy it would be to accomplish each change. To help them with adherence, they were asked to write down potential barriers that could prevent them from accomplishing each change. For each barrier, they were then asked to write down a strategy that would help them overcome this barrier. Participants were encouraged to adhere to the suggestions every day during the following month. To make this easier, they received a printable checklist to check off their adherence to changes on a daily basis. They also had an option to define their own small change they wanted to make in addition to the three suggestions and could choose to receive weekly reminders.

At the beginning of the following month, participants were sent an email inviting them back to the website, where they completed additional questions and were assigned new suggestions or tips for the subsequent month. The process repeated itself every month. Study procedures were approved by the Institutional Review Board.

### Participants

Participants were voluntary individuals who registered on the National Mindless Eating Challenge website between July 2007 and June 2009 and gave their consent for researchers to use their data for the purposes of the study (n=2053). The characteristics of all registered participants and returning participants (those who completed at least one follow-up survey) are presented in Table 1. The proportion of returning participants was 25% (504/2053). The returning participants were slightly older, more educated, and weighed slightly less than nonreturning participants (those who never returned for follow-up surveys after registration). Nonreturning participants were excluded from outcome analyses.

**Table 1:** Baseline characteristics of participants.

Characteristics		All participants (n=2053)	Returning participants (n=504)	*F* test, returning & nonreturning (*P* value)
**Age (years)** ^**a**^		39.8 (12.80)	42.6 (12.08)	32.737 (< 0.001)
**Female** ^**b**^		1829 (89)	458 (91)	1.215 (0.270)
**White/Caucasian** ^**b**^		1840 (90)	463 (92)	3.608 (0.058)
**United States** ^**b**^		1672 (81)	410 (81)	0.004 (0.951)
**College degree** ^**b**^		1641 (80)	423 (84)	6.667 (0.010)
**Household income < $50,000** ^**b**^		558 (27)	114 (23)	3.673 (0.055)
**Weight (lbs)** ^**a**^		172.2 (42.28)	168.9 (37.80)	4.119 (0.043)
**Body mass index** ^**a**^		28.1 (6.51)	27.9 (6.24)	1.030 (0.310)
**Initial eating goal** ^**b**^				
	Lose weight	1709 (83)	455 (88)	0.601 (0.438)
	Maintain weight	106 (5)	24 (5)	0.219 (0.639)
	Eat healthier	197 (10)	30 (6)	10.262 (0.001)
	Help family eat better	37 (2)	5 (1)	2.478 (0.116)

^a^ Values are expressed as mean (SD).

^b^ Values are expressed as n (%).

In addition to the United States, participants were from Canada (11%), the United Kingdom (2%), Australia (0.5%), Germany (0.5%), France (0.5%), and 32 other countries. Most participants (83%) had weight loss as their initial eating goal. Ten percent wanted to eat healthier, 5% wanted to maintain their weight, and 2% aimed to help their family eat better. Four participants did not specify whether they wanted to lose or maintain weight. Eating healthier was slightly more common as an initial eating goal among nonreturning than returning participants.

### Measures

Participant retention was measured by the number of monthly surveys participants completed in the program between July 2007 and July 2009 and by the number of calendar months participants stayed in the program (months that passed from the registration to the last completed follow-up survey).

All measures about participant characteristics were self-reported during registration or during follow-up surveys. Demographics (age, gender, race, education level, annual household income, and country) were asked in the registration survey. Weight and height were asked in the registration survey and in each follow-up survey.

Weight loss outcomes were calculated as the difference between the weight reported at the last follow-up survey a participant completed and the weight reported in the registration survey. Hence, the length of the follow-up varied between participants.

Adherence to habit change suggestions was measured as the number of days (0-31) participants reported having followed the suggestions they had been given. Perceived effectiveness of changes was measured on a 1-9 scale (Not Very Effective – Very Effective). The total amount of effective changes for each month was calculated as the number of changes that were rated as 6 or above in effectiveness. Participants’ experiences with changes were collected through free-form entries in follow-up surveys.

### Analyses

Descriptive statistics were used to characterize participant retention. Student *t* tests were performed to assess the overall significance of weight changes over time. Analyses of variance were used to compare the adherence to changes and the perceived effectiveness of changes between participants who lost, maintained, or gained weight between subsequent surveys. The suggestions with high adherence were examined by taking a subset of cases where at least 20 participants had reported adherence of at least 20 days. Student *t* tests were used to examine the significance of weight changes associated with suggestions with high adherence. The suggestions that participants considered as the most and the least effective were derived based on the mean effectiveness ratings of suggestions that had been received by at least 25 participants (approximately 5% of the sample). Demographic differences in tip perceptions were assessed with analyses of variance.

Reported experiences with changes were analyzed with qualitative content analysis methods. The experiences were categorized into main themes of barriers and facilitators, under which findings were further categorized under emerging subthemes. The total occurrences of themes were counted to identify recurring themes.

All quantitative analyses were done using SPSS version 19.0. *P* values less than .05 were considered statistically significant.

## Results

### Participant Retention

**Figure 1:**
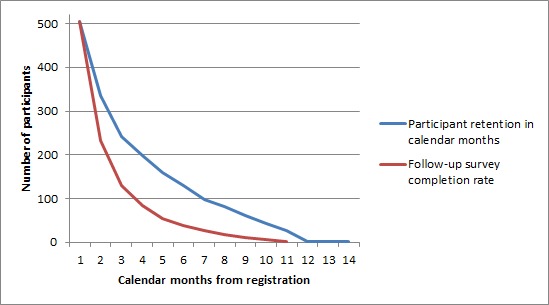
Participant retention and follow-up survey completion rate.

Figure 1 shows the adherence to the program over the course of the 14 months after signing up. Participant attrition was 75% after the initial registration: 1549/2053 participants never completed the intake survey or never returned for a follow-up survey. The participants who returned for at least one follow-up stayed in the program on average 3.7 calendar months (SD 3.10) and completed on average 2.2 follow-up surveys (SD 1.93). Most of them (88%, 445/504) had weight loss as their initial eating goal. Out of the returning participants, 38% (189/504) stayed in the program for more than two months and completed at least two follow-up surveys.

### Weight Changes

Over the course of the program, 42% of returning participants (213/504) lost weight (mean 3.24% of initial weight, SD 2.94), 29% (145/504) gained weight (mean 3.35%, SD 3.68), and 27% (136/504) maintained their weight over the course of the program. Weight change data were missing from 2% (10/504) of the participants. Overall mean weight loss was 0.41% (0.75 lbs) of the initial weight (*t*=-2.346, *P*=.019). Participants who had weight loss as their initial goal lost on average 0.48% (0.9 lbs) of their initial weight (*t*=-2.534, *P*=.012). Clinically significant weight loss, 5% or more of initial body weight, was achieved by 7% of the participants (36/504).

Table 2 presents the weight and BMI changes of participants with different levels of engagement in the program. The participants who stayed in the program for at least three months and completed at least two follow-up surveys (38% of the returning participants) lost on average 1.0% (1.8 lbs) of their initial weight (*t*=-2.622, *P*=.009). The mean time these participants stayed in the program was 6.4 months (SD 2.77), and they completed on average 4.0 follow-up surveys (SD 2.20).

**Table 2:** Weight and BMI changes among returning participants.

	Level of engagement		
	One-time visitors^a^	Two-month participants^b^	Three+ month participants^c^
Number of participants	271	44	189
% of returning participants	54	9	38
Mean weight change, lbs (SD)	-0.06 (5.746)** *P*=.868	-0.69 (3.982)** *P*=.263	-1.77 (8.574)** *P*=.006
Mean weight change, % (SD)	-0.04 (3.156)** *P*=.853	-0.38 (2.306)** *P*=.285	-0.97 (5.012)** *P*=.009
Mean BMI change (SD)	-0.09 (1.892)** *P*=.471	-0.01 (0.664)** *P*=.900	-0.26 (1.511)** *P*=.023

^a^ Completed only 1 follow-up survey.

^b^ Completed 1-2 follow-up surveys and stayed in the program for 2 months.

^c^ Completed at least 2 follow-up surveys and stayed in the program for at least 3 months.

### Adherence to Changes

**Figure 2:**
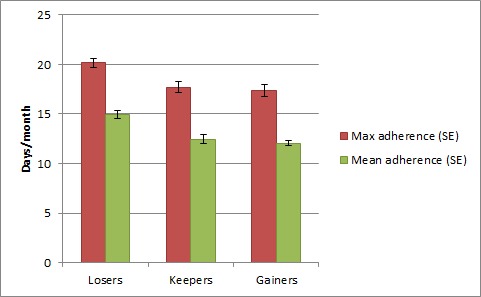
Adherence to three changes among participants who lost, maintained, or gained weight between any two surveys.

Adherence to changes was reported in 88% (979/1107) of all follow-up surveys. The days the participants reported having adhered to the habit change suggestions were on average 13.3 days (SD 9.77) over 1 month. Participants who had lost weight between subsequent surveys reported higher monthly adherence to suggestions (mean 14.9 days, SD 7.92) than participants who had maintained their weight (mean 12.4 days, SD 7.63) or who had gained weight (mean 12.0 days, SD 7.50; *F*=14.17, *P*<.001); see Figure 2. Similarly, maximum adherence was highest among weight losers.

### Adherence and Weight Outcomes

**Figure 3:**
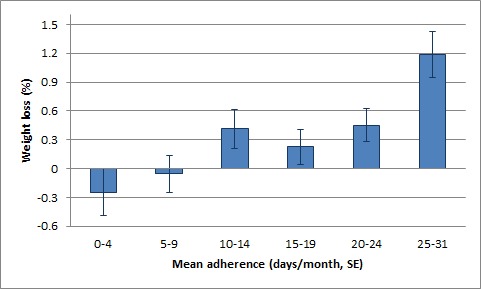
Percentage weight loss for different levels of adherence.

Participants who reported consistent adherence (at least 25 days in a month) to the suggested changes reported an average monthly weight loss of 2.0 lbs (*P*<.001). Figure 3 displays the percentage weight loss for different levels of mean adherence to suggestions. Participants whose mean adherence was 25 days or more had a mean weight loss of 1.2%, a significantly higher number than participants who adhered only 0-4 days (*F*=3.991, *P*=.001) or 5-9 days (*P*=.014). Mean adherence to suggestions was positively correlated with weight loss percentage (*r*=.166, *P*<.001). Moreover, adherence to a suggestion was correlated with perceived ease (*r*=.622, *P*<.001).

Table 3 presents the mean weight outcomes of a subset of cases in which suggestions had adherence reports of at least 20 days from at least 20 participants. Two suggestions in this subset of 14 suggestions were associated with significant weight loss and one on borderline significance.

**Table 3:** Weight outcomes of suggestions with high adherence.

Tip	n of cases	Mean weight change, lbs (SD)	*t* test (*P* value)	Mean adherence (SD)	Mean effectiveness (SD)	Mean ease (SD)
Put down your utensils between bites. (This will allow you to slow down the pace of your eating.)	23	-2.48 (3.85)	-3.089 (.005)	24.96 (4.14)	7.70 (1.64)	6.48 (2.09)
Allow yourself an afternoon snack only if you’ve first eaten a piece of fruit.	24	-1.88 (4.46)	-2.062 (.051)	24.42 (4.02)	7.00 (1.96)	6.86 (1.93)
Any time you think you might eat when you’re not hungry, go ahead and do so, but only if you first say (out loud): “I’m not hungry, but I’m going to eat this anyway”.	20	-1.58 (2.94)	-2.400 (.027)	22.90 (2.90)	6.05 (2.09)	6.00 (2.08)
Drink 8 cups of water a day (that’s only two full 32-oz glasses).	39	-1.29 (5.33)	-1.509 (.140)	24.32 (3.74)	6.20 (2.39)	7.10 (1.93)
Have a glass of water with every meal and snack.	30	-1.23 (5.75)	-1.173 (.250)	25.47 (4.13)	7.57 (1.83)	7.52 (1.68)
Use the Half-plate Rule: at dinner, load up the right side of your plate with salad, fruit, or vegetables. The other side can be starches and meat.	20	-1.05 (3.46)	-1.359 (.190)	24.05 (3.68)	7.20 (1.51)	7.10 (1.37)
Restrict your eating to the kitchen or dining room. (Doing this will make it more inconvenient to mindlessly eat between meals.)	24	-0.91 (4.02)	-1.107 (.280)	24.33 (3.97)	6.21 (2.59)	6.50 (2.23)
Eat something hot for breakfast at home within the first hour of waking up.	25	-0.79 (3.03)	-1.306 (.204)	26.16 (3.34)	6.92 (2.58)	7.42 (2.15)
Avoid going more than 3-4 hours without have something small to eat. (That way, you will be less likely to overdo it at meals.)	43	-0.75 (6.87)	-0.711 (.481)	25.51 (4.01)	6.69 (2.02)	6.93 (2.00)
Avoid eating anything directly from its bag, container, etc.	27	-0.66 (4.34)	-0.790 (.437)	24.67 (4.19)	6.89 (2.17)	6.30 (2.30)
Use smaller plates on meals.	21	-0.35 (2.98)	-0.542 (.594)	24.41 (3.91)	7.73 (1.16)	7.36 (1.99)
Never eat directly from a package − always portion food out into a dish so you need to face exactly what you will eat.	21	0.27 (4.81)	0.257 (.800)	23.71 (3.64)	7.19 (1.81)	6.86 (2.33)
Pack a baggie of precut veggies and fruit for at least one snack per day.	22	0.46 (3.69)	0.590 (.561)	23.87 (3.76)	7.17 (1.95)	7.17 (1.72)
Keep counters clear of all foods but the healthy ones.	21	0.86 (5.17)	0.760 (.456)	27.00 (3.46)	6.86 (2.24)	8.29 (1.49)

### Perceived Effectiveness of Changes

The average amount of suggestions that the returning participants perceived as effective was 1.46 (SD 1.06). The average perceived effectiveness of all suggestions was 5.12 (SD 2.73) on a 1-9 scale. Table 4 presents the five most effective and five least effective suggested changes. The table also displays the total numbers of participants who received the suggestion as well as the mean values for ratings of effectiveness and ease, reported adherence, and weight changes from the time the suggestion was received by a participant to the time of the follow-up.

Participants who lost weight between subsequent surveys reported a higher amount of effective suggestions (mean 1.66, SD 1.03) than participants who maintained weight (mean 1.38, SD 1.03) or gained weight (mean 1.24, SD 1.07; *F*=15.256, *P*<.001). Effectiveness was strongly correlated with adherence (*r*=.610, *P*<.001) and ease (*r*=.691, *P*<.001).

Some demographic differences were found in participants’ perceptions of suggestions. The mean effectiveness ratings for suggestions were higher among participants who were white/Caucasian (5.2 vs. 4.7, *F*=5.162, *P*=.023) or had at least a college degree (5.2 vs. 4.8, *F*=6.336, *P*=.012). Moreover, the mean ease ratings were higher among participants who were white/Caucasian (4.9 vs. 4.4, *F*=4.573, *P*=.033) or who were from the United States (4.9 vs. 4.6, *F*=4.070, *P*=.044).

### Barriers and Facilitators for Changes

Experiences of changes were reported in 745 follow-up surveys. Common barriers and facilitators for changes that emerged from the reported experiences are summarized in Tables 5 and 6. The identified barriers were roughly divided into change-related, personal, and external barriers. The most common change-related barrier was that the suggestion was in some ways unsuitable for the participant: for example, too specific to certain situations, actually making the problem worse, or inconvenient to do. In addition, several participants stated that some changes were not applicable to their lifestyles at all or that they were just difficult to implement in most situations. Within personal barriers, simply forgetting to make the changes and being too busy to pay attention to changes were the most common ones. Emotional eating (due to negative emotions, tiredness, or stress) and losing track or motivation (“I did not even try”) also came up often. The most commonly mentioned external barrier was unusual circumstances when eating behavior was less under one's own control (such as vacations or staying with someone else).

Facilitators for lifestyle changes were divided into program-related and personal facilitators. The most prevalent statement was that changes were “easy”. This statement was not usually elaborated further. Other program-related facilitators were reminders (calendar checklist, email reminders, or concrete environmental cues) and goal-setting. Personal facilitators were mostly related to gradual changes in awareness or behaviors and the feelings these changes evoked. Many participants commented that specific changes were less important than becoming aware of eating habits and paying attention to behaviors that had been mindless. Positive feelings as well as noticing results (such as enjoying food more and having energy) were other common themes.

**Table 4:** Most and least effective suggestions.

Suggestions		n of cases	Mean effectiveness (SD)	Mean ease (SD)	Mean adherence (SD)	Mean weight change, lbs (SD)
**Most effective**						
	1. Keep counters clear of all foods but the healthy ones.	31	6.8 (2.01)	7.6 (1.80)	23.8 (7.22)	0.3 (4.69)
	2. Never eat directly from a package – always portion food out onto a dish so you need to face exactly what you will eat.	52	6.7 (2.13)	5.9 (2.62)	16.1 (7.93)	-0.2 (3.70)
	3. Eat something hot for breakfast at home within the first hour of waking up.	42	6.3 (2.82)	6.6 (2.89)	20.0 (9.83)	-0.2 (3.56)
	4. Avoid going more than 3-4 hours without have something small to eat. (That way, you will be less likely to overdo it at meals.)	90	6.2 (2.35)	6.2 (2.49)	18.1 (9.04)	-0.8 (5.57)
	5. Put down your utensils between bites. (This will allow you to slow down the pace of your eating.)	72	6.1 (2.64)	4.7 (2.58)	13.5 (9.62)	-1.7 (5.29)
**Least effective**						
	1. Cinch your belt up 1 notch tighter than usual before you start to eat.	33	3.1 (2.79)	3.2 (2.86)	7.5 (9.65)	-0.9 (2.48)
	2. Brush your teeth when you feel like snacking (10:30 and 3:45 are the most tempting times).	48	3.4 (2.68)	3.6 (2.67)	6.3 (7.61)	0.2 (2.64)
	3. Use the 3 Bite Rule: eat whatever you want, but limit it to 3 small/medium-sized bites.	55	3.6 (2.32)	3.1 (2.27)	7.9 (7.53)	-0.8 (3.02)
	4. Exercise at a time when you usually snack. (This way you are not only removing calories that you would have normally eaten, you are also burning calories.).	26	3.8 (2.27)	3.6 (2.40)	7.2 (6.07)	-1.3 (2.46)
	5. After dinner, brush and floss your teeth to prevent evening snacking.	47	3.9 (2.71)	3.7 (2.76)	8.6 (7.95)	-0.5 (3.21)

**Table 5:** Common barriers to changes based on participants’ experiences.

Barrier		Prevalence	Common explanations
**Change-related barriers**			
	Unsuitable changes	87	Too specific (9), dislike (9), problematic to fit in the schedule (8), made problem worse (6), changes were incompatible (5), already a habit (5), wasting food felt difficult (5), irrelevant (4), inconvenient (3)
	Inapplicable changes	37	Situation not encountered (21), did not fit the schedule (4)
	Difficult changes	34	Difficult to do outside home (12), too much effort (7), difficult month (4), hard to plan ahead (4), hard to be consistent (2)
**Personal barriers**			
	Forgetting	83	Distractions (9), simply forgetting about changes
	Being busy	49	Lack of time (11), stress (11), busy schedule (5), major deadline (2)
	Not even trying	31	Lack of motivation (10), not feeling committed (5)
	Losing track	31	Losing motivation (11), no regular tracking (11), losing focus (8)
	Need to eat	30	Hunger (10), cravings (8), danger times (7), availability of food (6), overeating (5)
	Emotional eating	17	Stress eating (5), compulsive eating (2)
	Ingrained habits	14	Falling back into old patterns
**External barriers**			
	Unusual circumstances	57	Vacation (18), lack of control over food choices (14), traveling (12), holiday season (11)
	Health issues	18	Own (12), sickness (5), family (1)
	Social pressure	13	Partner’s/family’s habits, social gatherings
	Unavailability of food	11	Healthy food not at hand (5), no access to healthy food (3), fruit not in season (2)

**Table 6:** Common facilitators of changes based on participants’ experiences.

Facilitator		Prevalence	Common explanations
**Program-related facilitators**			
	Easy	75	Creating habits that can last (8), small change to existing habits, simple changes
	Reminders	21	Checklist and other concrete reminders (12), email reminders (5), environmental cues (4), accountability (4)
	Having goals	17	Thinking about goals (5), determination (3), strategies (3), regular tracking (3)
**Personal facilitators**			
	Increased awareness of eating habits	41	What, how, and when one eats, recognizing mindless eating habits
	Positive feelings	28	Not feeling deprived (6), not feeling hungry (6), enjoyment of food (3), feeling better (3)
	Modifying or expanding the changes	19	Continuing with earlier changes (6), making additional changes (3)
	Changes in eating habits	19	Eating more slowly (8), portion control (7), mindful eating (2)
	External support	15	Mindless Eating book (5), other health program (4), availability of healthy food (3), social support (2)
	Seeing results	13	Improvement from small changes (5)
	Planning ahead	11	Learning to plan and prepare
	Already a habit	9	Easy to increase frequency
	Psychological changes	9	Overcoming food-related issues (3), sense of control (2)

## Discussion

In this study, we evaluated weight outcomes and participant retention in a publicly available web-based healthy eating and weight loss program based on a small-changes approach. The results of the study showed significant but modest weight loss outcomes, with larger effects among participants who were more engaged in the program, stayed in it for a longer time, and completed more follow-up surveys. That is, those who completed at least three months of the program or adhered at least 25 days per month to the suggested changes reported a significantly higher average monthly weight loss than those who dropped out early or who did not adhere to the suggested changes. The small-changes approach shows promise, but encouraging adherence and finding suitable changes for each person still remain a challenge.

### Participant Retention

One fourth of the participants who registered and received the first set of habit change suggestions returned for follow-up. Loss of participants over time was fairly quick, with only half of those who returned for follow-ups staying in the program for more than two months. This kind of high attrition is typical for voluntary online programs, in which the intervention is neither mandatory nor critical to participants [18-21] and that do not provide additional incentives other than positive feedback and benefits to health and well-being. Attrition rates in weight loss interventions vary considerably even in face-to-face settings, with reported rates ranging from 10% to more than 80% [22]. In the case of NMEC, we can only speculate the reasons for participant attrition. We could propose three main reasons why participants stopped returning for follow-up: 1) they were satisfied with the results, 2) they decided that the program was not worth their time anymore, or 3) they just forgot about it while going on with their busy lives. It is likely that the main contributor is a decrease in motivation after the initial interest [10]. In addition, email reminders were the only method of communication with participants, and there was no real human contact that could have resulted in higher engagement to the program [21].

Nevertheless, rapidly decreasing retention is not necessarily an indication of the program failing to reach its aims. It has been suggested that the main role for web-based programs in prevention and treatment of obesity may be to deliver short positive messages and reminders that can lead to increased awareness and seeking of assistance from other sources [23]. The participants of the NMEC program may have needed the initial boost to get started with concrete habit changes, but after the initial month or two, some voluntarily reported that they had already gained enough awareness and skills to start making up their own changes that would best suit their individual circumstances. The strength of the small-changes approach is that the principle is simple and quick to learn [1,5]. Additionally, it is possible that some participants decided to acquire the book that was referred to in the program and felt no need to return to the online program after reading it. The book and the online program could be viewed as complementary self-help resources. In fact, it might be beneficial for participants if intervention programs contained references to external resources based on their needs as an alternative to combining treatment strategies for comorbidities into the same intervention [24]. For example, if there is a reason to suspect that a participant suffers from depression or anxiety, a weight loss program could guide them to interventions that handle such issues.

### Weight Outcomes and Effectiveness of Changes

Nearly half of participants lost weight over the course of the program, and the average amount they lost was 3.2% of their initial weight. Although the other half of participants either maintained or gained weight and the overall mean weight loss was modest, the results suggest that small-changes approach is promising in weight loss and maintenance, considering that effect sizes in online healthy eating and weight loss interventions have been generally small [9,25]. Moreover, most participants were overweight, not obese, and the focus of the program was not primarily losing weight but rather healthier and more mindful eating. Small weight losses or even maintenance of current weight are valuable achievements and useful in preventing weight gain [12]. High adherence was associated with larger outcomes: for those whose adherence to changes was 25 or more days per month, weight loss averaged 2.0 lbs in a month.

Half of the suggestions in the program were generally perceived as effective, and participants who lost weight rated a higher amount of suggestions as effective. Some tips that were reported as effective were associated with small (although not statistically significant) weight gain. This may have been due to other factors, but it may also indicate that people perceive effectiveness in different ways. Tips that were associated with weight gain or weight maintenance were likely to either increase the amount of healthy food consumed (“keep counters clear of all foods but the healthy ones”) or give a good start to each day (“eat something hot for breakfast”). Therefore, effectiveness could have meant that participants succeeded in changing the habit, ate healthier, and felt better about themselves even if they did not lose weight. This notion was supported by several participants’ comments.

Effectiveness, ease, and adherence were all strongly correlated. Hence, finding relevant and easy habit changes for each individual would be essential. Tailoring interventions to individuals generally increases effectiveness [7,8]; the NMEC program tailored suggestions simply based on participants’ eating goals. Further tailoring to individual circumstances and psychological characteristics would likely improve outcomes and adherence, and participants’ own predictions about ease and effectiveness of habit changes should be used to screen out changes that have a very low probability to succeed. Moreover, suggestions in the NMEC program were considered somewhat more effective and easy by white/Caucasian participants, more effective by those with higher education level, and easier by Americans. Because suggestions were developed based on research done in the United States, suggestions and the program itself may have been more suitable or attractive for an audience with similarities to the developers. Cultural tailoring in terms of language, graphics, and consideration of common eating habits and environments could increase participant adherence and satisfaction [26], although the most basic suggestions are likely to be widely applicable even without tailoring.

### Adherence to Changes

Not surprisingly, participants who lost weight adhered more to suggested changes than participants who maintained or gained weight. Even though the difference was small (a couple of days more per suggestion), it may be enough to tip the scale to the side of weight loss. Adherence was also strongly correlated with perceived effectiveness and ease, which suggests that no matter what the changes were, participants benefited from them if they committed to making them and found them easy to do. These findings are in line with earlier research that has associated higher intervention adherence with better behavioral outcomes [20,25]. If adherence to actual changes is low, the intervention does not have a lot of chance to impact behavior, except in the rare cases in which the impact results from keeping the goals in mind.

Considering that high adherence was associated with higher weight loss, identifying the best suggestions for weight loss could be possible by analysis of suggestions that received high adherence ratings. Among the 14 suggestions that were adhered to for at least 20 days by at least 20 participants, 2 were associated with significant weight loss. Both of them required some willpower but did not restrict the amount of eating or food choice; rather, they drew attention to eating pace or eating choices. Indeed, several participants commented that these kinds of suggestions helped to increase awareness of eating habits. Even though data about prior history of dieting were not collected in the program, several female middle-aged participants may have had earlier unsuccessful dieting experiences [3]. Many diets are characterized by restrictive rules that may lead to feelings of deprivation [17,27], binge eating [28], or eating bouts [29]. A small-changes approach could result in healthier attitudes towards food and eating in response to hunger and satiety signals since it does not restrict eating but makes people more conscious of their eating habits, if they are able to adhere to changes.

Adherence is likely to be mediated by the strength of the existing habits that need to be changed: if a new habit is supposed to replace an existing strong habit, the change is likely to be more difficult than if the habit to be replaced is weak or nonexistent [6,30]. This came up in several participants’ comments about deeply ingrained habits. Difficulty of a habit change influences how much time it will take to form a new habit. Lally and colleagues did a study with 96 participants and found that habit formation took on average 66 days, but there was a large variation from 18 days to 254 days depending on the complexity of the habit [31]. In the NMEC program, some participants said that they would have wanted to continue with the changes from the prior month rather than receive new suggestions. This may indicate that they were still struggling with habit formation or that they had been in unusual circumstances where changes were not applicable. Ideally, suggested changes should be generic and flexible enough so that they are doable every day. As some participants mentioned, this will provide a sense of accomplishment, improve self-efficacy, and encourage them to continue with further changes [32].

### Barriers and Facilitators for Changes

Analysis of participants’ experiences with changes indicates that habit change suggestions were perceived as more effective and easy to adhere to if they matched participants’ personal situation, lifestyle, and psychological needs. Unsuitable, inapplicable, or difficult changes were soon discarded as requiring too much effort or being irrelevant. Furthermore, unusual circumstances such as vacations and busy schedules with deadlines made it difficult to adhere to suggestions that concerned environmental changes and food choices, especially if the suggestions were situation-specific. To accommodate people’s changing circumstances such as travels and holiday seasons that disrupt existing habits [30], it may be most beneficial to provide flexible heuristics that are applicable to any situation. Another possibility is to attempt to profile participants’ needs frequently and adapt the advice for changing situations [33,34].

Losing track of changes or forgetting them completely was relatively common among participants. Email reminders and a calendar checklist helped several to monitor their behaviors and stay on track, but not everyone benefited from periodic prompts and reminders, which is in line with earlier studies [35]. Concrete cues and reminders in the environment, such as having the checklist in the kitchen, appeared to be helpful for several participants. Participants’ adherence to daily changes might be improved by encouraging and advising them to set concrete but unobtrusive triggers and cues in places where they can frequently see them [6,36]. The simple small-changes intervention could also lend itself ideally to mobile phones, which are carried around most of the day and accessed frequently.

Having easy changes to make, having goals in mind, and learning to plan ahead were helpful for participants [32], and suggestions that increased their awareness about their eating habits appeared to be especially useful. Such suggestions typically involved either modifying their eating environment or learning to focus and slow down. These kinds of suggestions could be used to overcome emotional eating, which was a fairly common stumbling block. A lot of needless eating in today’s society is caused by emotional needs that cannot be fulfilled, and some people use food instead to fill the emotional void or to fight their tiredness or stress [37]. Indeed, depression and obesity have been shown to have a reciprocal link [38]. Addressing the problematic relationship with food may require additional strategies that focus on improving self-esteem, self-control, and constructive coping [38].

### Limitations

The voluntary setting with no active recruitment or promotion of the program is both a limitation and strength of this study. That is, the program involved no human contact, and participants reported their own weight and their adherence. The results should be interpreted with caution because all measures were self-reported. Weight in subsequent surveys could have been reported on different times of the day or different weekdays, which can mask small actual changes in weight. Furthermore, there was no control group and participant attrition was high. Since only 25% of participants who registered to the website returned to the follow-up surveys, it is possible that the intervention effect is overestimated. Yet even in the absence of a control group, in this kind of a setting, the behavior of the participants was likely to resemble behavior of ordinary users of online weight loss and healthy eating programs; some of the people who registered may have just been curious and had no serious intention to start the program. Moreover, since data were collected across 2 full years, the results are generalizable across seasons and cannot be explained by seasonality (ie, people might lose more weight over the summer or gain more over the holidays). In the general population, all reports of changing weight point to a general increase and not a decrease [39-42].

In the analyses of the most and the least effective suggestions, the potential influence of the other suggestions that participants received cannot be ruled out. The pool of different suggestions was so large that only a relatively small number of participants received individual suggestions, which limits the possibilities to identify significant differences. To discover the most suitable and effective tips for different individuals, further studies would be needed.

### Conclusions

This study illustrates that an online intervention based on a small-changes approach can help individuals lose weight, especially if they adhere to changes consistently. Participants who were adherent to their suggested changes 25 or more days per month reported an average loss of 2 lbs each month. What is not fully known is how long this rate of slow and steady weight loss would continue. In general, adherent participants who continued past the 3-month mark lost a small but significant proportion of their weight. It’s important to note that these people were self-selected and may be much more diligent or motivated than the average person who joins a small-change nutrition and weight loss program.

High attrition remains a challenge that can potentially be solved with further tailoring to individual needs and tighter connection to participants’ everyday lives. For instance, asking more detailed screening questions during the initial profiling survey could provide more tailored suggestions and increase perceived relevance and anticipated adherence. Ensuring that changes are easy and require little effort from participants provides them opportunities to experience success and increased awareness of their eating habits and benefits of healthy eating, motivating them to continue on the chosen path. In addition, encouraging participants to place concrete cues and reminders in their environment could work even better than notifications through email or mobile devices. Such changes in a person’s food environment could lead them to become slimmer by design [43].

Long-term follow-up is needed to evaluate the maintenance of habit changes and weight loss. Of particular interest would be to better predict how likely a participant would be to adhere to a particular suggestion. Being able to better predict adherence could lead to more relevant and effective advice. Further research could also expand the small-changes approach to other important health behaviors such as physical activity or stress management.

## Multimedia Appendix 1

The Mindless Eating Challenge home page.

## References

[1] Hill JO (2009). Can a small-changes approach help address the obesity epidemic? A report of the Joint Task Force of the American Society for Nutrition, Institute of Food Technologists, and International Food Information Council. Am J Clin Nutr.

[2] Verplanken B, Wood W (2006). Interventions to break and create consumer habits. Journal of Public Policy & Marketing.

[3] Mata J, Todd PM, Lippke S (2010). When weight management lasts. Lower perceived rule complexity increases adherence. Appetite.

[4] van't Riet J, Sijtsema SJ, Dagevos H, De Bruijn GJ (2011). The importance of habits in eating behaviour. An overview and recommendations for future research. Appetite.

[5] Wansink B (2010). From mindless eating to mindlessly eating better. Physiol Behav.

[6] Rothman AJ, Sheeran P, Wood W (2009). Reflective and automatic processes in the initiation and maintenance of dietary change. Ann Behav Med.

[7] Krebs P, Prochaska JO, Rossi JS (2010). A meta-analysis of computer-tailored interventions for health behavior change. Prev Med.

[8] Noar SM, Grant Harrington N, Van Stee SK, Shemanski Aldrich, R (2011). Tailored health communication to change lifestyle behaviors. American Journal of Lifestyle Medicine.

[9] Webb TL, Joseph J, Yardley L, Michie S (2010). Using the internet to promote health behavior change: a systematic review and meta-analysis of the impact of theoretical basis, use of behavior change techniques, and mode of delivery on efficacy. J Med Internet Res.

[10] Cugelman B, Thelwall M, Dawes P (2011). Online interventions for social marketing health behavior change campaigns: a meta-analysis of psychological architectures and adherence factors. J Med Internet Res.

[11] Wieland LS, Falzon L, Sciamanna CN, Trudeau KJ, Brodney S, Schwartz JE, Davidson KW (2012). Interactive computer-based interventions for weight loss or weight maintenance in overweight or obese people. Cochrane Database Syst Rev.

[12] Arem H, Irwin M (2011). A review of web-based weight loss interventions in adults. Obes Rev.

[13] Neve M, Morgan PJ, Jones PR, Collins CE (2010). Effectiveness of web-based interventions in achieving weight loss and weight loss maintenance in overweight and obese adults: a systematic review with meta-analysis. Obes Rev.

[14] van Genugten L, van Empelen P, Boon B, Borsboom G, Visscher T, Oenema A (2012). Results from an online computer-tailored weight management intervention for overweight adults: randomized controlled trial. J Med Internet Res.

[15] Poirier J, Cobb NK (2012). Social influence as a driver of engagement in a web-based health intervention. J Med Internet Res.

[16] Wansink B, Just DR, Payne CR (2009). Mindless Eating and Healthy Heuristics for the Irrational. American Economic Review.

[17] Wansink B (2006). Mindless eating: why we eat more than we think.

[18] Eysenbach G (2005). The law of attrition. J Med Internet Res.

[19] Glasgow RE, Nelson CC, Kearney KA, Reid R, Ritzwoller DP, Strecher VJ, Couper MP, Green B, Wildenhaus K (2007). Reach, engagement, and retention in an Internet-based weight loss program in a multi-site randomized controlled trial. J Med Internet Res.

[20] Donkin L, Christensen H, Naismith SL, Neal B, Hickie IB, Glozier N (2011). A systematic review of the impact of adherence on the effectiveness of e-therapies. J Med Internet Res.

[21] Brouwer W, Kroeze W, Crutzen R, de Nooijer J, de Vries NK, Brug J, Oenema A (2011). Which intervention characteristics are related to more exposure to internet-delivered healthy lifestyle promotion interventions? A systematic review. J Med Internet Res.

[22] Moroshko I, Brennan L, O'Brien P (2011). Predictors of dropout in weight loss interventions: a systematic review of the literature. Obes Rev.

[23] Christensen H, Mackinnon A (2006). The law of attrition revisited. J Med Internet Res.

[24] Linde JA, Simon GE, Ludman EJ, Ichikawa LE, Operskalski BH, Arterburn D, Rohde P, Finch EA, Jeffery RW (2011). A randomized controlled trial of behavioral weight loss treatment versus combined weight loss/depression treatment among women with comorbid obesity and depression. Ann Behav Med.

[25] Cugelman B, Thelwall M, Dawes P (2011). Online interventions for social marketing health behavior change campaigns: a meta-analysis of psychological architectures and adherence factors. J Med Internet Res.

[26] Barrera M, Castro FG, Strycker LA, Toobert DJ (2012). Cultural adaptations of behavioral health interventions: A progress report. J Consult Clin Psychol.

[27] Booth DA, Jarvandi S, Thibault L (2012). Food after deprivation rewards the earlier eating. Appetite.

[28] Polivy J, Herman CP (1999). Distress and eating: why do dieters overeat?. Int J Eat Disord.

[29] Wansink B (1994). Antecedents and Mediators of Eating Bouts. Family and Consumer Sciences Research Journal.

[30] Verplanken B, Wood W (2006). Interventions to break and create consumer habits. Journal of Public Policy & Marketing.

[31] Lally P, van Jaarsveld CHM, Potts HWW, Wardle J (2010). How are habits formed: Modelling habit formation in the real world. European Journal of Social Psychology.

[32] Anderson-Bill ES, Winett RA, Wojcik JR (2011). Social cognitive determinants of nutrition and physical activity among web-health users enrolling in an online intervention: the influence of social support, self-efficacy, outcome expectations, and self-regulation. J Med Internet Res.

[33] Collins LM, Murphy SA, Bierman KL (2004). A conceptual framework for adaptive preventive interventions. Prev Sci.

[34] Honka A, Kaipainen K, Hietala H, Saranummi N (2011). Rethinking health: ICT-enabled services to empower people to manage their health. IEEE Rev Biomed Eng.

[35] Fry JP, Neff RA (2009). Periodic prompts and reminders in health promotion and health behavior interventions: systematic review. J Med Internet Res.

[36] Papies EK, Hamstra P (2010). Goal priming and eating behavior: enhancing self-regulation by environmental cues. Health Psychol.

[37] Kemp E, Bui M, Grier S (2010). Eating their feelings: Examining emotional eating in at-risk groups in the United States. Journal of Consumer Policy.

[38] Van Dorsten B, Lindley EM (2011). Cognitive and behavioral approaches in the treatment of obesity. Med Clin North Am.

[39] Flegal KM, Carroll MD, Ogden CL, Curtin LR (2010). Prevalence and trends in obesity among US adults, 1999-2008. JAMA.

[40] Ford ES, Li C, Zhao G, Tsai J (2011). Trends in obesity and abdominal obesity among adults in the United States from 1999-2008. Int J Obes (Lond).

[41] Wang Y, Beydoun MA, Liang L, Caballero B, Kumanyika SK (2008). Will all Americans become overweight or obese? Estimating the progression and cost of the US obesity epidemic. Obesity (Silver Spring).

[42] von Ruesten A, Steffen A, Floegel A, van der ADL, Masala G, Tjønneland A, Halkjaer J, Palli D, Wareham NJ, Loos RJ, Sørensen TI, Boeing H (2011). Trend in obesity prevalence in European adult cohort populations during follow-up since 1996 and their predictions to 2015. PLoS One.

[43] Wansink B (2013). Slim by Design.

