# Supplementary figures and images for: Mindless Eating Challenge: Retention, Weight Outcomes, and Barriers for Changes in a Public Web-Based Healthy Eating and Weight Loss Program
Source: J Med Internet Res. 2012 Dec 17;14(6):e168. doi: 10.2196/jmir.2218 (PMC3799612; doi:10.2196/jmir.2218)

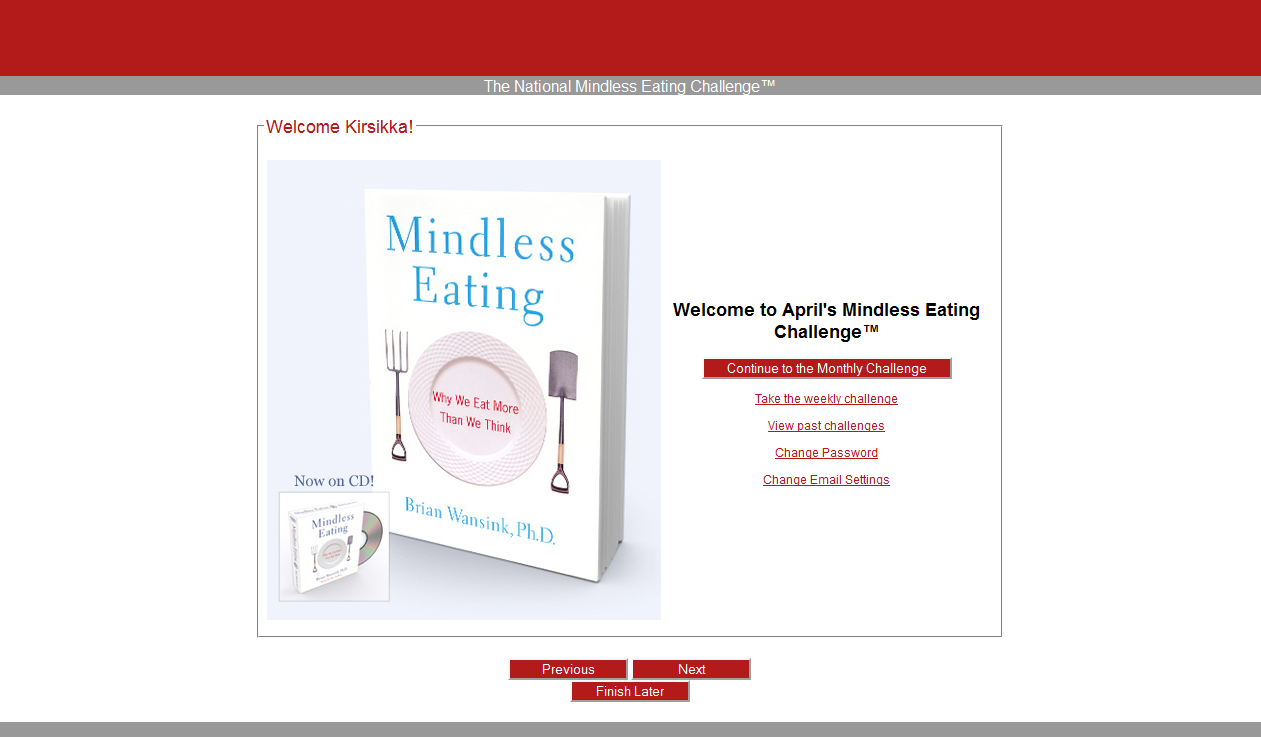

Supplement: Supplementary file 1 [file jmir_v14i6e168_app1.png]
